# *N*-acetyl-β-D-glucosaminidase in clinically healthy German Shepherd dogs and dogs with early stages of chronic kidney disease

**DOI:** 10.17221/34/2024-VETMED

**Published:** 2024-10-31

**Authors:** Nikola Marecakova, Jana Kacirova, Csilla Tothova, Aladar Madari, Marian Madar, Maria Kuricova, Slavomir Hornak

**Affiliations:** ^1^Small Animal Clinic, University Veterinary Hospital, University of Veterinary Medicine and Pharmacy in Kosice, Kosice, Slovak Republic; ^2^Institute of Plant Genetics and Biotechnology, Plant Science and Biodiversity Centre, Slovak Academy of Sciences, Nitra, Slovak Republic; ^3^Clinic of Ruminants, University Veterinary Hospital, University of Veterinary Medicine and Pharmacy in Kosice, Kosice, Slovak Republic; ^4^Department of Microbiology and Immunology, University of Veterinary Medicine and Pharmacy in Kosice, Kosice, Slovak Republic

**Keywords:** enzyme activity, kidney disease, urinalysis, urine enzyme, tubular marker

## Abstract

Urinary *N*-acetyl-β-D-glucosaminidase (NAG) activity is associated with kidney disease. In our study, we focused on evaluating the ratio of NAG to the urinary creatinine concentration (NAG/Cr) in the German Shepherd breed. Sixty-two healthy dogs and thirteen dogs with chronic kidney disease (CKD) were examined. The healthy dogs were divided into groups based on their sex and age (puppies and adolescent dogs under 2 years, adult dogs from 2 to 6 years and seniors over 6 years), while the dogs with CKD were divided based on the disease stage (CKD stage 1 and 2). No significant difference was detected between the males and females. Regarding the age dependence, significantly higher values were found in dogs older than 6 years (*P* = 0.020 3) compared to dogs aged 2–6 years. When comparing the healthy dogs to the dogs with CKD, the lowest NAG/Cr ratio was observed in the dogs with CKD stage 2. The reference interval for NAG/Cr in the German Shepherd was established in the range of 0.78–7.86 U/gCr. We observed no correlations with the commonly used markers of kidney disease such as creatinine, urea, or symmetrical dimethylarginine. Nevertheless, we encourage the further investigation of NAG in relation to CKD, taking the breed, sex and age of the patients into account.

Current conventional diagnostic tests for kidney damage in the blood (serum creatinine, urea and symmetrical dimethylarginine) and urine (urine protein creatinine ratio, urine specific gravity and urine sediment) are widely used for the diagnosis and monitoring of kidney disease (Braun et al. 2003). The measurement of the glomerular filtration rate (GFR) provides the most accurate assessment of the renal function and is the most sensitive method for the early detection of kidney dysfunction. However, many clearance tests are costly and time-consuming and not suitable for widespread use as a routine screening test (Ghys et al. 2014). A low activity of urinary enzymes is observed in healthy animals, and finding an increase in their activity indicates a kidney injury (Tassini et al. 2018).

*N*-acetyl-β-D-glucosaminidase (NAG) is a high molecular-weight (∼140 kDa) tubular lysosomal enzyme recognised as a marker of tubular damage, with tubular damage causing the release of NAG and the consequent increase in enzyme activity in urine (Sabbisetti and Bonventre 2011; De Loor et al. 2013). Several studies have shown an increase in NAG in dogs with chronic kidney disease (CKD), which is thought to be due to the tubular damage or increased lysosomal change secondary to proteinuria (Sato et al. 2002; Smets et al. 2010a; Nabity et al. 2012). The strong correlation with glomerular damage and insufficient correlation with tubular damage support the possibility that NAG may cross the damaged glomerular filtration barrier (Hokamp and Nabity 2016). Therefore, although NAG is used to detect tubular damage in acute renal injuries, it may also be useful for detecting glomerular damage in chronic proteinuric nephropathy.

NAG has been shown to be a predictor of future renal failure in non-albuminuric, non-azotaemic human patients with type-II diabetes who developed diabetic nephropathy over a 5-year period (Okazaki et al. 2005). In a study focusing on cats, the NAG index was positively correlated with the urine protein-creatinine (UPC) ratio and was significantly higher in cats that developed azotaemia than in those remaining non-azotaemic within 12 months. However, it was not retained within the multivariable, leading us to question whether the NAG index has any major additional benefit to the measurement of proteinuria in predicting the development of azotaemia (Jepson et al. 2009).

The urinary NAG-to-creatinine ratio (NAG/Cr) is increased in dogs with chronic kidney disease, pyelonephritis, uncontrolled diabetes mellitus, pyometra or X-linked hereditary nephropathy. However, the NAG/Cr did not change significantly in dogs with adrenocorticotropic hormone (ACTH)-dependent hyperadrenocorticism at risk of renal dysfunction before and after treatment with trilostane or transsphenoidal hypophysectomy (Sato et al. 2002; Smets et al. 2010a; Nabity et al. 2012; Smets et al. 2012). The aim of this study was to determine the NAG activity in healthy German Shepherd dogs and in dogs with early stages of CKD. We also focused on the possible influence of age and sex on the NAG/Cr in a population of healthy German Shepherd dogs.

## MATERIAL AND METHODS

### Sample animals

In total, 75 dogs were included in the study. The animals were handled humanely. All the applicable international, national and institutional guidelines for the care and use of animals were followed. The clinical examinations and sampling were performed with the informed consent of the dog owners. The population of 62 healthy German Shepherd dogs (0.3–12 years old) were working dogs, kept in the same conditions and fed the same food. The dogs were divided into groups according to age (puppies and adolescent dogs up to 2, adult dogs 2–6 years old, and seniors over 6 years old). Both males (*n* = 25) and females (*n* = 37) were intact, the females were not pregnant or lactating. At the time of examination, all the dogs had no signs of disease, their blood and urine analysis were without any pathological findings, the animals were normotensive (VET HDO; S+B medVet, Babenhausen, Germany) and ultrasonographic examination of the abdominal cavity (ProSound Alpha 6; Hitachi Aloka Medical Ltd., Tokyo, Japan) was without any pathological changes.

Thirteen German Shepherds were diagnosed with CKD according to the serum creatinine and symmetric dimethylarginine (SDMA) concentration. These dogs were further subclassified by measuring the level of proteinuria and the presence of hypertension following the IRIS classification (www.iris-kidney.com/guidelines/staging.html). Overall, seven dogs were included into CKD stage 1 group, and six dogs into CKD stage 2 group based on the anamnesis, clinical examination, imaging methods, and laboratory examination.

### Sampling and laboratory analysis

After a fasting period of at least 12 h, blood samples were collected from the *vena cephalica antebrachii* into a tube without an anticoagulant. The samples were centrifuged at 1 500 *g* for 10 min and then the serum was subjected to a complete biochemical profile (AST, ALT, GGT, ALP, CK, Chol, Lip, Amyl, Crea, Urea, TBil, Glu, TP, Alb, P, Ca, K, Na and Cl) using the Cobas C 111 (Roche, Basel, Switzerland) and the SDMA was tested using the Catalyst One (IDEXX Laboratories, Westbrook, ME, USA). The haematology was analysed from unclotted blood, taken into a test tube with tetra potassium ethylenediaminetetraacetic acid (K4EDTA), using the ProCyte Dx (IDEXX Laboratories, Westbrook, ME, USA).

Urine was collected by ultrasound-guided cystocentesis. Subsequently, the urine samples were centrifuged at 700 *g* for 5 min and subjected to a complete routine urinalysis, which included a physical examination, measurement of the urine specific gravity using a refractometer, a semiquantitative chemical examination with DekaPhan Leuko diagnostic strips, and a microscopic analysis of the urine sediment. Proteinuria was quantified by the ratio of protein to creatinine in the urine (UPC) measured on a Cobas C 111 instrument (Roche, Basel, Switzerland). The collected urine was also sent for urine culture on nutrient media to detect any urinary infection.

The urine samples were stored at 4 °C and the enzymatic activity was determined within 72 h of collection without the freezing and thawing of the samples. The NAG activity was measured using a commercial canine specific sandwich enzyme-linked immunosorbent assay ELISA (MBS005370; MyBioSource Inc., San Diego, CA, USA) following the manufacturer’s instructions. Concisely, the standards (100, 50, 25, 12.5, 6.25 and 3.12 U/l) and urine samples were applied to the corresponding wells and horseradish peroxidase-conjugate reagent was added. The microtiter plate was incubated at 37 °C for 60 min, followed by manual washing. After adding chromogen solution A and chromogen solution B, the plate was again incubated at 37 °C for 15 minutes. Finally, the stop solution was added and the optical density of each well was determined at a wavelength of 450 nm within 10 min using an Opsys MR automatic microplate reader (Dynex Technologies, Chantilly, VA, USA). The urinary NAG activity was expressed as units per litre (U/l) and, to minimise the effect of the urine specific gravity, was calculated to the NAG/Cr ratio (U/gl).

### Statistical analysis

Data processing and statistical analysis were performed using GraphPad Prism v9.4.0 statistical software (GraphPad Software, San Diego, USA). A principal component analysis (PCA) was conducted with the determined biomarkers (creatinine, urea, SDMA, urinary creatinine and urinary NAG) as the variables. An unpaired *t*-test with Welch’s correction or a one-way analysis of variance (ANOVA) with an additional Tukey’s test was used to determine the statistical differences between the healthy dogs of different sexes and ages, but also between the healthy dogs and the dogs with CKD. The interactions between the analysed parameters of the healthy dogs and the dogs with CKD were evaluated using Pearson’s correlation analysis at *P* < 0.05.

The calculation method for the reference interval with respect to the sample size, its descriptive statistics and the normality of the distribution was parametric with a logarithmic transformation. Firstly, we identified and removed outliers using the ROUTH method, Q = 1%. Statistical tests for the occurrence of (potential) outliers and for normality were set at a 5% significance level and the reference intervals were set at 95% and the confidence intervals for the limits were set at 90%.

## RESULTS

A total of 75 German Shepherds were included in the study, the dogs were divided based on the IRIS classification into three groups (healthy, CKD1 and CKD2). The haematology and complete biochemistry profile (data not shown), concentration of creatinine, urea, SDMA, urinary creatinine and NAG was determined for each dog (Table 1).

**Table 1:** General information about the dogs and the results of the laboratory analyses

General information	Healthy	CKD1	CKD2
Average age (years) Range	3.8 0.3–12	5.2 1.2–9	4 2–9
Male (*n*)	25	4	3
Female (*n*)	37	2	4
Crea (μmol/l); RI < 125	79.12 ± 14.32	107.40 ± 20.86	132.26 ± 13.50
Urea (mmol/l); RI: 3.97–8.05	5.70 ± 1.63	7.27 ± 2.20	9.12 ± 1.38
SDMA (μg/l); RI < 140	86.27 ± 19.64	75.00 ± 39.37	115.71 ± 58.27
UCrea (mmol/l)	17.69 ± 8.25	22.64 ± 10.35	23.73 ± 6.49
NAG (U/l)	48.63 ± 13.28	48.29 ± 8.06	49.23 ± 8.42

Values are presented as mean ± standard deviation

CKD1 = chronic kidney disease stage 1; CKD2 = chronic kidney disease stage 2; Crea = serum creatinine; NAG = *N*-acetyl-β-D-glucosaminidase; RI = reference interval; SDMA = symmetric dimethyl arginine; UCrea = urinary creatinine

**Figure 1:**
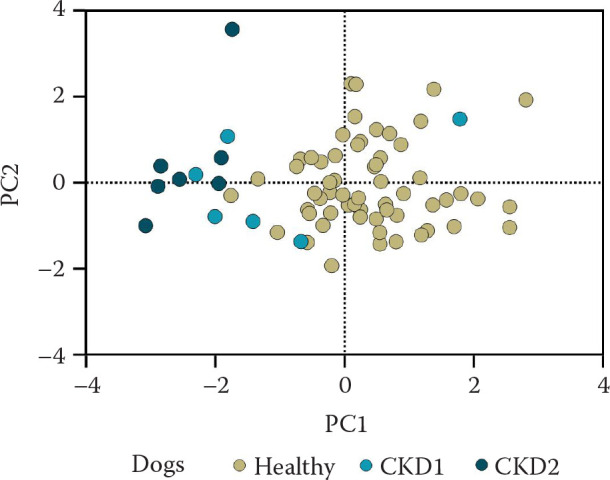
Principal components analysis (PCA) of the biomarkers (creatinine, urea, symmetric dimethylarginine, urinary creatinine and urinary *N*-acetyl-β-D-glucosaminidase) Principal components with the largest eigenvalues of healthy dogs, dogs with chronic kidney disease stage 1 and stage 2 (CKD1 and CKD2). Each dog is represented by an individual point

With the determined renal biomarkers (creatinine, urea, SDMA, urinary creatinine and urinary NAG) as the variables, the PCA allowed the visualisation of how the individuals clustered within the groups with respect to their principal components compressed from the data. The PCA revealed a separation between the healthy dogs and the dogs with CKD. PC1 explains 33.90% of the variance and PC2 explains 21.80% of the variance (Figure 1).

**Figure 2:**
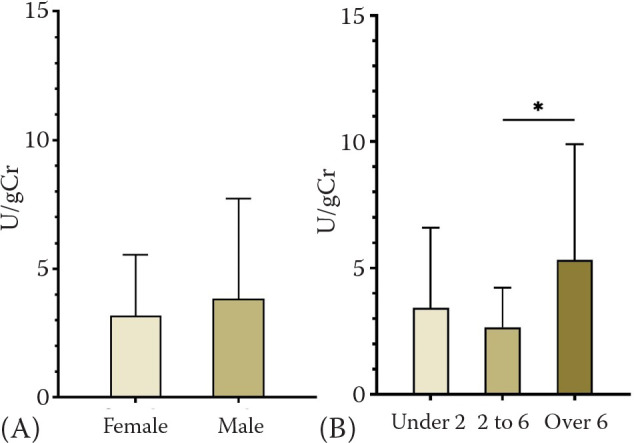
Concentration of the NAG/Cr in healthy dogs divided by sex (A) and age (B) Age is given in years. Data are presented as mean ± standard deviation. The asterisks indicate significant differences between the groups analysed by the one-way ANOVA with Tukey’s test (*P ≤ 0.05)

To eliminate the effect of the urine specific gravity, the ratio of NAG to creatinine (NAG/Cr) was calculated. In the group of healthy German Shepherd dogs (*n* = 62), the obtained results were compared on the basis of their sex and age. There was a statistically significant difference in the NAG/Cr values between the groups based on the age (*P* = 0.020 3) (Figure 2B), but no statistically significant differences were found based on the sex (Figure 2A). The lowest values were determined in the adult dogs aged 2–6 years, while the highest values were determined in the dogs older than 6 years.

**Figure 3:**
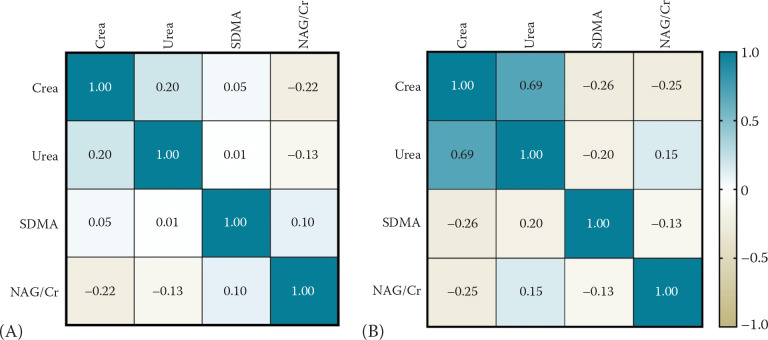
Heatmap of Pearson’s correlation between the NAG/Cr and kidney markers in the healthy dogs (A) and in the dogs with CKD (B) Values are R (correlation coefficient) NAG/Cr = urinary *N*-acetyl-β-D-glucosaminidase to creatinine ratio; SDMA = symmetric dimethyl arginine

The interactions between the NAG/Cr and the other parameters commonly used to diagnose kidney disease were determined. However, no significant correlation was found between the NAG/Cr and urea, creatinine or SDMA in the healthy dogs or in the dogs with CKD also (Figure 3A,B). The only positive correlation, between the creatinine and urea, was observed in the CKD group.

**Figure 4:**
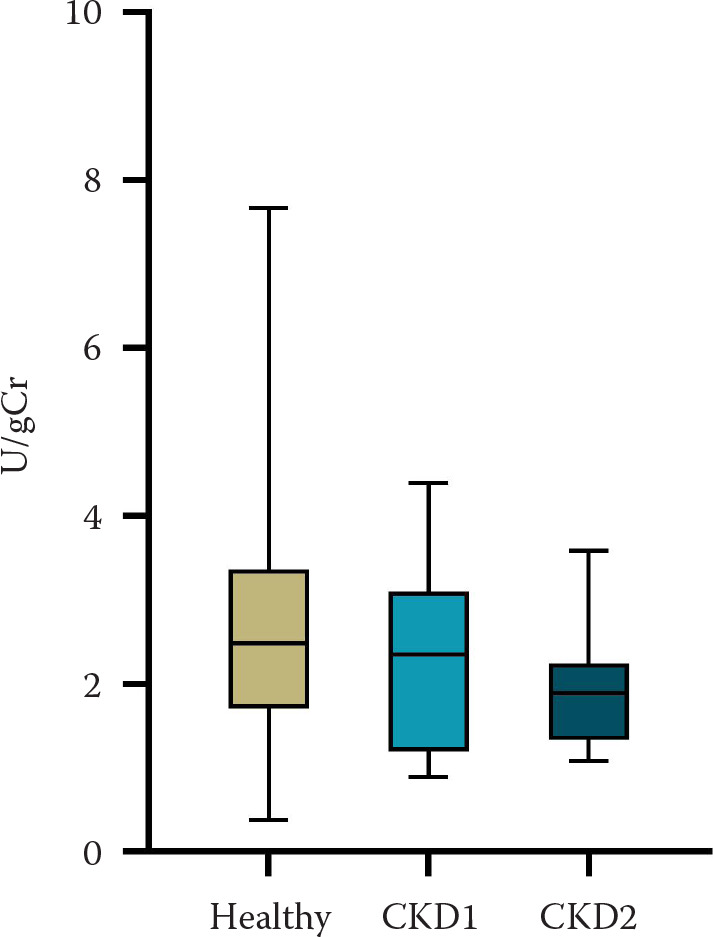
Comparison of the NAG/Cr concentration in the healthy dogs and the dogs with chronic kidney disease after the removal of the outliers in the group of healthy dogs No outliers were detected in the dogs with CKD CKD1 = chronic kidney disease stage 1; CKD2 = chronic kidney disease stage 2

In the group of healthy dogs, three outliers (10.78, 11.88 and 18.83) were identified and removed, subsequently the reference interval (RI) was determined to be 0.78–7.86 U/gCr. When comparing the healthy dogs (*n = *59) and the dogs with CKD, the mean value of the NAG/Cr in the CKD stage 2 group (2.01 U/gCr) was lower than in the CKD stage 1 group (2.32 U/gCr) and in the healthy dog group (2.92 U/gCr). However, no significant differences were noted (Figure 4).

## DISCUSSION

Despite many years of research for acute and chronic kidney diseases, reliable early disease biomarkers are still lacking. Potential biomarkers include glycosidases, enzymes involved in carbohydrate metabolism. The study by Tassini et al. (2018) determined the reference interval for the urinary NAG activity in the urine of healthy dogs of different breeds, sexes and ages (3 months – 15 years old). An automated method was used to determine the NAG activity standardised for dog urine samples. Based on the determination of the urinary activity and after correcting the values for a 1.025 urine specific gravity, the reference interval X’ = 3.62 U/l ± 0.66 U/l was obtained. In a study by Brunker et al. (2009), which focused on different breeds of dogs aged 1 to 5 years, the reference range of NAG/Cr was established to 0.02–3.63 U/g. While, in the study by Sato et al. (2002), in which dogs aged 1 to 8 years were included, NAG/Cr was 3.2 ± 2.4 U/g. In our study, we established a reference interval for NAG/Cr in the range of 0.78–7.86 U/gCr in healthy German Shepherds. Further investigation of breed variations is necessary even with a higher number of individuals in certain categories.

Through the statistical analysis, NAG was found to be positively correlated with the animal’s age and urine specific gravity, but not affected by the sex or weight (Tassini et al. 2018). We did not find significant gender differences in the urinary NAG excretion between the female and male dogs, which is also confirmed in humans (Agirbasli 1996; Pennemans et al. 2013), while Nakamura et al. (1983) found, in male Beagle dogs, a NAG activity approximately double that in females which was 2.4-fold higher when the activity was expressed relative to the urinary creatinine. Also, Brunker et al. (2009) demonstrated a significantly different urinary NAG index (NAG/Cr) between male and female dogs.

The increasing urinary NAG activity in older dogs over 6 years corresponds to the results in humans, while increased values were also observed in children at an early age (Olah et al. 2004). In the study by Pennemans et al. (2013), the trend for NAG was quadratic, with higher concentrations in both children and the elderly compared to 20–50-year-old subjects. This is mainly due to changes in muscle mass and, consequently in the creatinine excretion (Price 1992). A relatively frequent increase in the NAG/Cr in young and occasionally in older dogs was observed in a study by Nabiti et al. (2012). In contrast, no significant difference was detected between healthy young and older dogs (Smets et al. 2010a). The true effect of age is best assessed by a longitudinal follow-up of the same individuals.

Excessive amounts of urinary enzymes appear in the urine due to leakage from damaged tubular cells (Emeigh Hart 2005). Urinary NAG has proven to be a useful tool in the early detection of renal injury in various human diseases, as reported by Skalova (2005). In veterinary medicine, an increased NAG/Cr has been observed in dogs at various stages of CKD (Uechi et al. 1994; Sato et al. 2002; Smets et al. 2010a; Hokamp and Nabity 2016). In our study, on the contrary, the values of the ratio of enzyme activity to urinary creatinine concentration in dogs with CKD stage 2 were reduced compared to the healthy group and the CKD stage 1 group.

Small amounts of NAG are normally excreted in the urine, but tubular dysfunction greatly increases its excretion (Braun et al. 2003). Storage conditions can affect the urinary NAG activity, after storage, NAG was significantly lower compared with the fresh samples (Smets et al. 2010b). The samples in our study were never frozen and thawed, after collection, they were cooled and subsequently examined within 3 days. However, we cannot rule out the influence of the urine pH on the NAG activity. Several studies have examined the utility of urinary NAG for detecting CKD in dogs. Other studies revealed a large overlap of the NAG index values in healthy dogs and dogs with CKD (Smets et al. 2010a; Nabity et al. 2012; Tassini et al. 2018). An increased NAG activity has also been used to detect the onset of AKI associated with pyometra and leishmaniasis (Palacio et al. 1997). In addition, one study found increased NAG in dogs with poorly controlled diabetes mellitus, although it is possible that these dogs had nephropathy secondary to diabetes (Sato et al. 2002).

When using urine spot samples, the normalisation of the urinary enzyme activity-to-urinary creatinine does not preclude any within-day variation (Heiene et al. 1991). Enzymuria may be an excessively sensitive test to assess kidney injury when measured at only one time point. Therefore, the increase in activity of a single enzyme at one time point has limited diagnostic value (Clemo 1998). Assays have been validated for the species-specific measurement of urinary enzymes in dogs. However, these studies did not uniformly use the same validated assays, which complicates the direct comparison of the results. In addition, the daily variability and variations of biomarker concentrations must be taken into account before changes in the concentrations can be used as biomarkers in urine which can be correctly attributed to changes in kidney function (Uechi et al. 1994; Nabity et al. 2007). Changes in the creatinine production (e.g., due to changes in the muscle mass) or excretion must be taken into account when comparing the urinary creatinine and biomarker ratios of the same dog over time. Second, interindividual differences in the urinary creatinine have been described in dogs, allowing the overestimation or underestimation of renal dysfunction (Braun et al. 2003; De Loor et al. 2013).

Our study has some limitations. There was a lower number of animals in the groups of CKD dogs and males and females were not equally represented in the individual groups. However, by testing one breed, we wanted to avoid differences between breeds, as well as the influence of breeding and diet in this study. The cohort of dogs from our study were kept in the same conditions, fed the same food and all are active at work.

In conclusion, the aim of our study was to determine the urinary NAG enzyme activity in one breed and to compare the results in healthy dogs and dogs with the early stages of CKD. We found significantly higher values in old German Shepherd dogs over 6 years and lower values in dogs with CKD stage 2. Through our investigation, we concluded that NAG is a promising marker of kidney disease in the early stages. Further studies focused on the effect of age, sex or breed and also with a higher number of CKD patients compared to healthy individuals are needed, since our patients with CKD1 were mostly older, which could have an impact on the NAG activity.

## Conflict of interest

The authors declare no conflict of interest.
